# The Importance of WE in POWER: Integrating Police Wellness and Ethics

**DOI:** 10.3389/fpsyg.2020.614995

**Published:** 2020-12-18

**Authors:** Daniel M. Blumberg, Konstantinos Papazoglou, Michael D. Schlosser

**Affiliations:** ^1^California School of Professional Psychology, Alliant International University-San Diego, San Diego, CA, United States; ^2^Department of Criminal Justice, New Jersey City University, New Jersey, NJ, United States; ^3^Police Training Institute, University of Illinois at Urbana-Champaign, Champaign, IL, United States

**Keywords:** law enforcement, wellness, ethics, moral risks, resilience, training

## Abstract

In this article, the authors introduce the POWER perspective of police wellness and ethics. POWER stands for Police Officer Wellness, Ethics, and Resilience. The perspective represents the view that wellness and ethics cannot be discussed separately; they are inextricably connected to each other. Initiatives to address one should always, simultaneously, include the other. Although there is a need for wellness and ethics to be addressed on an organizational level, the present article emphasizes the importance of POWER for individual police officers. The authors make the argument that officers need to expand the way in which they conceptualize their own wellness to include efforts to maintain ethical decision-making. Specifically, officers will remain psychologically healthier when they take active steps to stay steadfastly committed to their ethical principles. Likewise, officers who utilize a comprehensive wellness program, including strategies to boost resilience, will be far less likely to experience lapses in ethical decision-making. Further recommendations for action and implication of this matter in law enforcement are presented and discussed.

For the past few decades, police officers' health and fitness has received increased attention by researchers as well as among police executives (Gilmartin, 2002; Mumford et al., 2015). The job is undeniably difficult and filled with a variety of physical, cognitive, emotional, and social stressors. Resilience has been identified as a mechanism that can mitigate the negative impacts that police officers experience from their job (Queirós et al., 2020). Similarly, for at least as long as officer wellness has been a concern, it has been a priority to most people who are concerned with police practices (i.e., social scientists, politicians, community members, and police executives) to understand and to curtail police corruption (Klockars et al., 2004). Many theories, which attempt to explain unethical decision-making, have been examined in relation to routine police practices (Blumberg et al., 2018).

Recently, a new viewpoint, which integrates these topics, has emerged (Papazoglou and Blumberg, 2020). In this article, the authors introduce the POWER perspective of police wellness and ethics. POWER stands for Police Officer Wellness, Ethics, and Resilience. The perspective represents the view that wellness and ethics cannot be discussed separately; they are inextricably connected to each other. Initiatives to address one should always, simultaneously, include the other. Although there is a need for wellness and ethics to be addressed on an organizational level (Blumberg et al., 2020; Thoen et al., 2020), the present article emphasizes the importance of POWER for individual police officers. The authors make the argument that officers need to expand the way in which they conceptualize their own wellness to include efforts to maintain ethical decision-making. Specifically, officers will remain psychologically healthier when they take active steps to stay steadfastly committed to their ethical principles. Likewise, officers who utilize a comprehensive wellness program, including strategies to boost resilience, will be far less likely to experience lapses in ethical decision-making (see Blumberg et al., in press).

Psychologically healthy police officers are more likely to stay committed to their ethical principles. However, the relationship between wellness and ethics may not be intuitively clear to officers or to their police leaders. This requires connecting some of the dots. Research has pointed out that police officers are significantly more likely than the general population to experience depression and anxiety (Mumford et al., 2015; Bergman et al., 2016). A noteworthy source of the depression and anxiety is emotional exhaustion caused by officers' job demands and job resources (Santa Maria et al., 2018). Additionally, the stress of policing can manifest in excessive levels of anger (Bergman et al., 2016). This is significant because anxiety and anger have been found to negatively influence ethical decision-making (Kligyte et al., 2013; Griffith et al., 2016; Zhang et al., 2020). Therefore, although police officers have little control over their job demands and job resources, it is important for them to focus on areas in which they do have considerable control; officers can develop strategies to combat emotional exhaustion and to prevent the occurrence of unhealthy levels of anxiety, depression, and anger.

Before discussing a few of these strategies, it should be mentioned that some of the job demands that negatively impact officers' emotional health also increase the likelihood that they will engage in misconduct (for an extensive review, see Blumberg et al., 2018). The job demands are organizational and operational. Organizationally, it begins during training:

Specifically, byproducts of the training may be (a) to foster us vs. them attitudes, (b) to instill strong bonds among (officers) in order to rely on each other to stay out of trouble or to avoid punishment, (c) to learn that there is a difference between the letter of the law and the spirit of the law (i.e., police officers often have to use discretion), and (d) to understand that morality is sometimes situational or relative, for example, police officers are legally permitted to lie to or deceive a suspect (Blumberg et al., 2016, p. 65).

After training, organizations may foster corruption of the noble cause whereby police leaders often condone police work in which the ends justify the means, like breaking rules to catch criminals (Crank et al., 2007; Loyens, 2014).

Operationally, there are numerous routine job demands that increase the likelihood that officers will engage in unethical decision-making. For example, officers' use of discretion means that they regularly make compromises by ignoring some crimes, while enforcing others. Likewise, when discretion is used without impartiality (e.g., who gets a ticket and who gets a warning), it indicates a lack of ethical decision-making. Perhaps the theory that best explains how policing fosters unethical decision-making is moral disengagement (Bandura, 1999), which describes eight mechanisms whereby individuals are disinhibited from acting unethically. Each of the eight mechanisms of moral disengagement occur during routine police work (Blumberg et al., 2018). Two of the mechanisms, dehumanization and attribution of blame, are particularly relevant when it comes to officers engaging in unethical behavior that will directly impact their wellness.

Police officers and the mental health experts who work with them need to be aware of the synergistic effect between police officers' ethical decision-making and their emotional health. As mentioned, officers who experience emotional difficulties are more likely to make unethical decisions. And, there are deleterious emotional effects when officers make unethical decisions through, for example, the mechanisms of moral disengagement, such as dehumanization and attribution of blame. Specifically, moral injury (Jinkerson, 2016) is a condition that leaves officers with feelings of guilt, shame, anger, and betrayal. Moral injury occurs when officers do something (or fail to do something) that violates their core values. For example, an officer is more likely to mistreat a dehumanized community member and to avoid helping someone who they blame for their own misfortune. Such actions can leave officers questioning their moral values, which results in feelings of guilt and shame. Moral injury also occurs when officers feel betrayed by the unethical actions of trusted colleagues or supervisors. The sense of betrayal leads to feelings of anger. Moral injury and the emotions of those suffering from it may be key factors in helping to understand and to curtail the skyrocketing rate of police suicide (Barr, 2020; Thoen et al., 2020).

The odds may seem stacked against police officers who are trying to stay emotionally healthy and ethically strong. However, many law enforcement agencies now provide psychological services that are available to their employees and a growing number of agencies have instilled a dedicated Wellness Unit (Creighton and Kaye, 2020). The psychological services tend to be geared toward intervention, as a resource for officers and their family members who seek some guidance, support, or psychotherapy. Although the Wellness Unit staff respond to critical incidents and support officers following traumatization, their goals include more prevention-based services as well (Creighton and Blumberg, 2016). Additionally, many law enforcement agencies have begun to implement wellness-based continuing education workshops and training classes for their officers. However, these efforts at an organizational level will not provide the lasting impact that police executives expect, unless there are corresponding procedural organizational improvements to ensure that officers utilize what they learn in these classes (Blumberg et al., 2020). Thus, police officers must take charge of their own wellness efforts. Rather than relying solely on training provided by their employer, officers can develop a commitment to lifelong learning and self-improvement. Ideally, this will include finding opportunities to improve their wellness and maintain their ethical commitments.

Research on officer wellness has shown promising results, and some of it directly addresses ethical decision-making as well. Several studies have demonstrated the efficacy of mindfulness-based stress reduction and/or resilience-boosting training. For example, a mindfulness-based resilience training reduced police officers' anger levels as well as their experience of organizational and operational stressors (Bergman et al., 2016). On a non-police sample, moral reasoning and ethical decision making improved following mindfulness-based stress reduction training (Shapiro et al., 2012). Also, in a very promising study, in only 4 4-h training sessions, significant improvements were observed in police officers' “emotional intelligence, empathy, resilience and stress management level,” and the changes remained at a 3-month follow-up (Romosiou et al., 2019, p. 11). In another study, officers' resilience and empathy improved in a six-session 8 week training (Blumberg et al., 2020). This is especially relevant, because of the inclusion of the emotional intelligence and empathy components of those trainings. People with higher emotional intelligence make fewer unethical decisions (Krishnakumar and Rymph, 2012). Also, emotional intelligence improves police officers' job performance (Ali et al., 2012). Furthermore, efforts to increase officers' empathy would be an excellent way to prevent the occurrence of moral disengagement (Bandura, 1999).

Police officers do not have to participate in formal training programs to stay healthy and ethically strong. A comprehensive self-help resource will soon be available (Blumberg et al., in press). However, on their own, police officers can simultaneously improve their wellness and ethical commitments with a few basic, integrated strategies. Of course, fitness and nutrition are important, along with good sleep hygiene and avoidance of self-medication, for physical health. When integrating wellness and ethics, the emphasis expands for officers first to focus on their spiritual health. Officers can learn and practice mindfulness or meditation. They can regularly engage in gratitude exercises. And, they can identify and frequently revisit their core values and life's purpose. Despite the many organizational pressures and operational challenges that officers face, when they attend to their spiritual wellness, it will be easier for officers to remain unwaveringly true to themselves.

Beyond spiritual wellness, it is important for officers to find ways to prevent some of the moral risks of policing that can impact their health and erode their commitment to ethical principles. This involves taking active steps to avert compassion fatigue (Figley, 1995), emotional exhaustion and burnout (Schaible and Gecas, 2010), and moral injury (Papazoglou et al., 2020). One strategy is for officers to utilize techniques that boost compassion satisfaction (Grant et al., 2019; Papazoglou et al., 2019; Millard, 2020). This is the sense of gratification that comes from helping others, especially those who have been victimized, and even those who are not particularly appreciative of the help. It requires officers to focus on the small wins and not to become burdened by the fact that they cannot help everyone. Additionally, when officers find ways to connect with and become part of the community in which they work (Schlosser, 2020), they minimize the likelihood of becoming morally disengaged. Less moral disengagement is vital to reducing the incidents that can lead to moral injury. A byproduct of community involvement is greater familiarity with members of the community, which will make officers more confident and comfortable (i.e., less anxious) when interacting with those community members. For that matter, anything that can make officers more confident and comfortable while performing their jobs will be beneficial to their health as well as advantageous to the community.

Another central tenet of the POWER perspective is that police officers are mere mortals even though they often do the work of superheroes. That means, like all humans, police officers will make mistakes and need to learn how to forgive themselves for an error. Unlike mistakes made by most people, however, some mistakes by officers can have tragic consequences. Ideally, the organization maintains a culture of wellness and ethics that supports officers after a mistake. However, regardless of the organizational response to the mistake (which can range from corrective counseling to termination), officers who strive to stay healthy have to learn to face their mistakes with humility and integrity. This requires the ability to examine why the mistake occurred. Was it from a lack of skill or a training deficiency? If so, then the officer should be motivated to improve the skill through some remediation. Was the mistake due to carelessness? If so, then the officer needs to explore what were the distractions and to take steps to increase mindfulness skills. Was the mistake due to anxiety, fear, or other emotions that interfered with performance? If so, then the officer should seek guidance to understand the source of the feelings and how to prevent them from hindering the work (e.g., cognitive-behavioral therapy). Without the willingness to critically examine one's performance and the capacity for self-forgiveness, wellness and ethical decision-making will become compromised.

The POWER perspective of wellness, ethics, and resilience helps law enforcement agencies and police officers to view wellness in an integrated way. Officers cannot stay healthy without maintaining a steadfast commitment to their core values. Similarly, when officers violate their moral code, their wellness will suffer. Initiatives to improve wellness should concurrently focus on officers' ethical decision-making. Likewise, efforts to reduce officers' misconduct must also address their physical, cognitive, emotional, spiritual, and social wellness. Although law enforcement agencies play a critical role in steps to improve officers' wellness and ethics, it is ultimately up to each individual police officer to incorporate a comprehensive wellness and ethics program.

## Data Availability Statement

The original contributions generated for the study are included in the article/supplementary material, further inquiries can be directed to the corresponding author/s.

## Author Contributions

DB created first draft and afterwards further revisions followed by KP and MS. All authors contributed equally in the development of ideas and structure of the manuscript.

## Conflict of Interest

The authors declare that the research was conducted in the absence of any commercial or financial relationships that could be construed as a potential conflict of interest.
